# Nanoscale Brownian heating by interacting magnetic dipolar particles

**DOI:** 10.1038/s41598-017-01760-x

**Published:** 2017-05-10

**Authors:** Yann Chalopin, Jean-Claude Bacri, Florence Gazeau, Martin Devaud

**Affiliations:** 1 Laboratoire d’Energétique Moléculaire et Macroscopique, CNRS UPR 288, CentraleSupelec, F-92295 Châtenay-Malabry, France; 2 Laboratoire Matière et Systèmes Complexes, UMR7057 CNRS/Universitè Paris Diderot, 76205 Paris, Cedex 13 France

## Abstract

Clusters of magnetic nanoparticles have received considerable interest in various research fields. Their capacity to generate heat under an alternating magnetic field has recently opened the way to applications such as cancer therapy by hyperthermia. This work is an attempt to investigate the collective effects of interacting dipoles embedded in magnetic nano-particles (MNP) to predict their thermal dissipation with a liquid. We first present a general approach, based on the tracking of the microscopic dipole fluctuations, to access to the dissipation spectra of any spatial distribution of MNPs. Without any other assumption that the linear response regime, it is shown that increasing the particle concentration (dipolar interactions) dramatically diminishes and blueshifts the dissipation processes. This effect originates in a predominance of the coupling energy over the Brownian torques, which create a long-range ordering that saturates the response of the system to an external field. Consequently, the particle density is of fundamental importance to the control of the absorption of electromagnetic energy and its subsequent dissipation in the form of heat.

## Introduction

Magnetic nanoparticles (MNPs) have been proposed in various applications for their ability to generate local heating of their environment by converting externally-supplied magnetic energy. The remote activation of particles by magnetic fields enables breakthrough approaches in biomedicine as well as catalysis and magnetic recording. MNP-mediated heating has been investigated as cell-destructive cancer therapy for decades^1^ and more recently for stimulation of thermosensitive nociceptive ion channels, neuronal modulation, deep brain stimulation, control of cell membrane depolarization, activation of protein expression or drug delivery^2–6^. A low-intensity radiofrequency alternating magnetic field can penetrate into the body without substantial attenuation allowing on-demand excitation of biocompatible iron oxide nanoparticles targeted to the desired area. Thermal dissipation in the environment depends on intrinsic properties of materials such as magnetization, anisotropy and size but also on the interactions of particles with the medium and other particles^7^. Particularly, when MNPs are encapsulated in drug vectors (liposomes, polymersomes) or embedded in biological environments (cell membrane, cell lysosome), they are submitted to dipole-dipole interactions that change their magnetic response to the external field in a complex and currently unsolved manner^8–10^. Going beyond the assumption of negligible interparticle interactions in the linear response theory is a burning issue, generally tackled using Monte-Carlo approaches^11–13^. Interparticle interactions are fundamental to many other disciplines including data storage^14^, geomagnetism, biology^2–6, 15^, composite materials, ferrofluids or magnetic resonance. The physics underlying the dynamics of interacting dipoles in systems of microscopic dimension is so rich that tremendous efforts have been devoted to address this subject from experimental^16, 17^ and theoretical^18^ approaches. Predicting the dynamics of the dipolar sets, comprising magnetic and dielectric nanostructures, is an important challenge as the long-range and anisotropic dipolar interactions^7^ entail a wealth of nonindependent degrees of freedom, difficult to track analytically. The developments of both numerical and analytical methods assessing the particles response to an external field^11–13^, and powerful enough to handle the complexity of the couplings, are still to be found. The particles are usually considered with a finite magnetic anisotropy and the computation of their relaxation properties relies on the energy barrier approximation^19^. In such cases, the relaxation is driven by two processes: The Neel and the Brownian relaxation. Regarding the former, many works have reported a shortening of the magnetization relaxation time when increasing the dipolar interactions^18–21^. In addition, various studies have further addressed the case of two-dimensional square arrays^20–26^ which are suited for experimental investigations^24^. In a similar fashion, dielectric relaxation in systems subjected to rotational Brownian motion remains an important question^27, 28^. It is relevant to the study of dynamic light scattering, Raman scattering and liquid crystals. Some analytical approaches have accounted for approximations in which the many-body system is casted in terms of a two-body problem^29^, or a single dipole in a mean field. The former approach is restricted to the case of low dipole densities while the latter is irrelevant for 2D or 1D sets.

Considering this broad setting, our interest focuses on the problem of evaluating the effect of the interactions between rigid dipoles carried by particles damped by a viscous torque^30^. Our analysis thus aims at identifying to what extent the Brownian relaxation process is affected by the particle interactions without any restriction on their density. For this purpose, we have formulated a statistical approach based on numerical simulations that reproduce the equilibrium orientational fluctuations of dipoles interacting in a heat bath. Hence, by means of the fluctuation-dissipation theorem, we have predicted the frequency-dependent imaginary part of the susceptibility of the system, subjected to an external ac field ${\vec{B}}_{1}$. We anticipate our conclusion by revealing that (1), interactions yield an additional relaxation rate and reduce the amplitude of susceptibility spectra and that (2), as the dipole pair energy grows, the magnetic stability of the system increases against the effect of the thermal agitation by the liquid, so that the absorption and the dissipation dramatically decrease.

## Model

We have considered a square array of *N* = 100 magnetic rigid dipoles ${\vec{\mu }}_{i}$ carried by spherical particles with volume *V* and inertia momentum *I*, immersed in a liquid with viscosity *η* at the temperature of 320 K. With the help of the fluctuation-dissipation theorem, it is possible to formulate a general approach to derive the macroscopic non-equilibrium response of the system by tracking numerically the microscopic equilibrium fluctuations of the dipoles ${\vec{\mu }}_{i}$. The Hamiltonian of the system can be written as

1 $$H=\frac{1}{2}\sum _{i,j=1}^{N}{U}_{ij}-\sum _{i=1}^{N}{\vec{\mu }}_{i}\cdot {\vec{B}}_{1},$$

considering the dipole-dipole coupling energy

2 $${U}_{ij}=\frac{{\mu }_{0}}{4\pi {r}_{ij}^{3}}[{\vec{\mu }}_{i}\cdot {\vec{\mu }}_{j}-3\frac{({\vec{\mu }}_{i}\cdot {\vec{r}}_{ij})({\vec{\mu }}_{j}\cdot {\vec{r}}_{ij})}{{r}_{ij}^{2}}],$$

where ${\vec{r}}_{ij}={\vec{r}}_{i}-{\vec{r}}_{j}$. The magnetization of the system is defined here as

3 $$\vec{M}=\sum _{i}{\vec{\mu }}_{i}\mathrm{.}$$

*A priori*, both the rotation and the translation^31^ of the particles are sources of dissipation. We nevertheless only consider the rotation of the magnetic dipoles in this study: the dipoles have a position fixed in a two-dimensional square lattice along the (*x*, *y*) plane, with the nearest neighbor distance *d*, entailing a dipole surface density *ρ* = 1/*d* ^2^. Each dipole ${\vec{\mu }}_{i}=\mu {\vec{e}}_{i}$ undergoes a viscous torque ${\vec{{\rm{\Gamma }}}}_{vis,i}=-\,\zeta {\vec{\omega }}_{p,i}$, with ${\vec{\omega }}_{p,i}$ the instantaneous rotation vector of dipole ${\vec{\mu }}_{i}$ and $\zeta =6\eta V$ the angular viscous friction coefficient. In addition to this damping, each dipole *i* is subjected to a magnetic torque ${\vec{\mu }}_{i}\times {\vec{B}}_{i}$ with

$${\vec{B}}_{i}={\vec{B}}_{1}-\frac{{\mu }_{0}}{4\pi }\sum _{j\ne i}({\vec{\mu }}_{j}\cdot {\vec{\nabla }}_{i})(\frac{{\mathop{r}\limits^{\longleftarrow}}_{ij}}{{r}_{ij}^{3}})\mathrm{.}$$

To account for the collisions with the liquid molecules, we have considered an angular Langevin equation with a stationary random torque ${\vec{{\rm{\Gamma }}}}_{rnd,i}$ acting on ${\vec{\mu }}_{i}$. The correlation time of this torque is consequently the collision time, which remains much smaller than the relaxation time of the angular velocity ${\vec{\omega }}_{p,i}$. ${{\rm{\Gamma }}}_{rnd}(t)$ is thus delta-correlated in time: $\langle {{\rm{\Gamma }}}_{rnd}(t){{\rm{\Gamma }}}_{rnd}(t+\tau )\rangle =2\zeta {k}_{B}T\delta (\tau )$. The complete set of differential equations describing the dynamics of the magnetic dipoles damped in a liquid is

4 $$\{\begin{array}{c}Id\overrightarrow{{\omega }_{p,i}}/dt={\overrightarrow{\mu }}_{i}\times {\overrightarrow{B}}_{i}-\zeta {\overrightarrow{\omega }}_{p,i}+{\overrightarrow{{\rm{\Gamma }}}}_{rnd,i}(t)\\ d{\overrightarrow{\mu }}_{i}/dt=\overrightarrow{{\omega }_{p,i}}\times {\overrightarrow{\mu }}_{i}\end{array}$$

In principle, it would be possible to study the response of the system illuminated by an external alternating field ${\vec{B}}_{l}(\omega )$ along direction *l* by including explicitly B_*i*_ in Eq. (4). The dynamics of a magnetic dipole in an oscillating field has been studied with a similar approach^32^. Practically, this procedure requires too many calculations to determine the whole spectrum of the magnetic susceptibility tensor *α*(*ω*) defined as:

5 $${\vec{M}}_{k}(\omega )={\alpha }_{kl}(\omega ){\vec{B}}_{l}(\omega \mathrm{).}$$

Instead, we propose to integrate Eq. (4) in the absence of external field to access to the equilibrium fluctuations of the rigid dipoles. Thanks to the fluctuation-dissipation theorem^33, 34^ (FDT), the components of the imaginary part of the magnetic susceptibility tensor *α*′′ = *Im*(*α*(*ω*)) can be obtained from the time correlation function of $\vec{M}$ as:

6 $${\alpha }_{kl}(\omega )=\frac{\langle {M}_{k}\mathrm{(0)}{M}_{l}\mathrm{(0)}\rangle }{N{k}_{B}T}+\frac{i\omega }{N{k}_{B}T}{\int }_{0}^{\infty }\langle {M}_{k}(t){M}_{l}\mathrm{(0)}\rangle {e}^{-i\omega t}dt,$$

with

7 $$\langle {M}_{k}(t){M}_{l}\mathrm{(0)}\rangle ={\int }_{-\infty }^{+\infty }{M}_{k}(t+\tau ){M}_{l}(\tau )d\tau ,$$

$k,l\in \{x,y,z\}$ and N is the number of particles considered. This approach has a significant advantage as the correlation function of the magnetization is trackable experimentally^35^. More importantly, from a single simulation, one thus predicts the spectral response of the system when excited by any small external field, by tracking the microscopic time fluctuations of the response without the field.

## Methods

Our simulations are based on the Langevin stochastic equations of motion (Eq. (4)) which are solved numerically using the Verlet method. The dipolar fluctuations are obtained in the canonical ensemble (fixed number of particles, constant volume and temperature) at T = 320 K. The effect of the rotational friction with the fluid is accounted for by a random force generated via a stochastic white noise, plus an effective friction force accounting for the water viscosity *η* = 7 · 10^−4^ Pa.s^−1^. The power spectral density of the random force Γ_*rnd*_ is related to the friction coefficient by the fluctuation-dissipation theorem^36^. The integration time step dt $\mathrm{=10}$ ps is large enough to capture the diffusion of the dipoles in a reasonable amount of time steps (typically ${10}^{8}$ steps), and sufficiently small to properly account for the characteristic times associated to the particle damping. We have evaluated the interactions by direct summation of the torques in the real space by means of a cutoff. This cutoff corresponds to twice the distance by which the dipole pair energy of a particle has converged, considering the highest density. A cutoff taken at 100 nm results in an error smaller than 0.02 k_*B*_T in estimating the magnetic energy per particle.

A summary of the simulation parameters is presented here on Table 1.

**Table 1:** Simulation parameters.

Parameter	value (units)
dt	10 (ps)
*η*	7 · 10^−4^ (Pa.s^−1^
T	320 (K)
Mass density	5 · 10^3^ (kg.m^−3^)
*μ*	1.3 · 10^5^(*μ*_*B*_)

## Results

**Figure 1:**
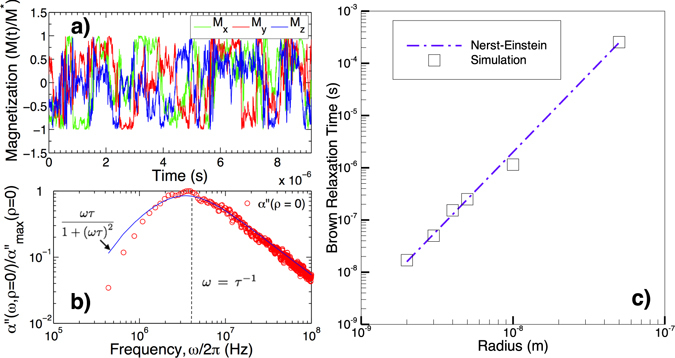
Noninteracting MNPs (**a**) Time fluctuations of the components of the magnetization $\vec{M}(t)$. *M*_*x*/*y*_ and *M*_*z*_ denote the in-plane and cross-plane components of the magnetization respectively. (**b**) Frequency dependence of the imaginary part of the susceptibility *α*(*ω*, *ρ* = 0) obtained according to Eq. (6), from the time fluctuations of $\vec{M}(t)$. The solid line shows the susceptibility fit with a Debye fonction (Eq. (8)) with a maximum for *ω*_*max*_ = 1/*τ*. (**c**) Relaxation time *τ* derived from the Debye fit of the susceptibility exemplified in (**b**), for different MNP radii (squares). The dashed line displays the Nernst-Einstein relation, in agreement with the simulations.

We first consider the case of noninteracting rigid dipoles. It corresponds to the limit where the inter-particle distance *d* → ∞, so that there is no effect of the dipolar interactions and *ρ* = 0. The dipoles are carried by spherical particles of various diameters. Figure 1 depicts the time fluctuations of the components of the magnetization $\vec{M}(t)$ and the imaginary part of the susceptibility spectra *α*′′(*ω*) extracted from these fluctuations following Eq. (6). As expected, it can be observed that *α*′′(*ω*) fits with a Debye law according to the formula

8 $$\alpha ^{\prime\prime} (\omega ,\rho =0)=\alpha (\omega =0,\rho =\mathrm{0)}\frac{\omega \tau }{1+{(\omega \tau )}^{2}}\mathrm{.}$$

**Figure 2:**
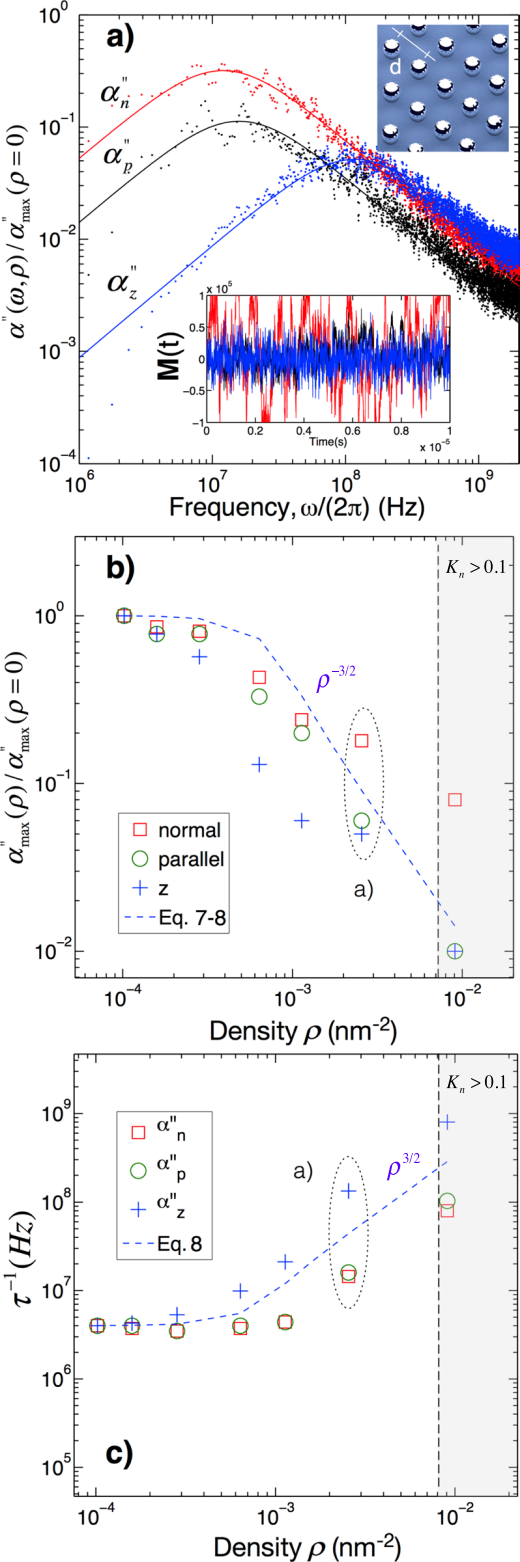
Interacting MNPs of 5 nm radius. (**a**) The inset illustrates the center-to-center inter-particle distance d. Imaginary part of the magnetic susceptibility *α*′′(*ω*, *ρ*) for *ρ* = 2.5 · 10^−3^ *nm* ^−2^ normalized by its maximum for noninteracting MNPs. The magnetic relaxation along the cross-plane direction $({\alpha }_{z}^{^{\prime\prime} })$ is weaker and faster than those of the in-plane components $({\alpha }_{n}^{^{\prime\prime} },{\alpha }_{p}^{^{\prime\prime} })$. The spectra obtained from a single equilibrium simulation (points) are fitted with a Debye function (solid lines). (**b**,**c**) Evolution of the susceptibilities ${\alpha }_{n,p,z}^{^{\prime\prime} }$ as a function of the MNP density. Dipole-dipole interactions diminish the absorption amplitude (**b**) and increase the frequency of maximum of dissipation (**c**). This effect is further enhanced along the cross-plane direction $({\alpha }_{z}^{^{\prime\prime} })$ while an in-plane component $({\alpha }_{p}^{^{\prime\prime} })$ is always more affected by the interactions than the other $({\alpha }_{n}^{^{\prime\prime} })$. The grey region indicates concentrations where hydrodynamics effects may occur due to small inter-particle distances defining a larger Knudsen number.

The lowest frequencies are attributed to the slowest characteristic times, thus, the longer one increases the simulation duration, the better one estimates the lowest frequency part of the spectra (in our case, *N*_*s*_ = 1^8^ steps with *dt* = 1 *ms*). At the optimal excitation angular frequency *ω* = *τ* ^−1^, the dissipation *α*′′(*ρ* = 0) reaches its maximum value ${\alpha }_{{\rm{\max }}}^{^{\prime\prime} }(\rho =\mathrm{0)}\equiv \alpha (\omega ={\tau }^{-1},\rho =0)=\alpha (\omega =0,\rho =0)\mathrm{/2}$, where *τ* is the relaxation time of the system (Fig. 1b). For MNPs with hydrodynamic diameters ranging from 5 nm to 50 nm, the simulations give relaxation times that quantitatively agree with the expected Nerst-Einstein relation *τ* = *τ*_*B*_ = *ζ*/2*k*_*B*_ *T* = 3*ηV*/*k*_*B*_ *T* (Fig. 1c). This result validates the statistical calculation in the well-known case of noninteracting rigid dipoles experiencing angular Brownian relaxation. Note that, as expected, the in-plane component of the susceptibility ${\alpha }_{n/p}^{^{\prime\prime} }$ (${\vec{e}}_{n}$ and ${\vec{e}}_{p}$ being any couple of orthogonal in-plane directions) and the cross-plane components of the susceptibility ${\alpha }_{z}^{^{\prime\prime} }$ are identical in this case. We have next included the dipole-dipole interactions between MNPs. The strength of the interactions is tuned with the particle surface density *ρ*. Figure 2b displays the anisotropic dissipation spectra for the two components ${\alpha }_{n}^{^{\prime\prime} }$ and ${\alpha }_{p}^{^{\prime\prime} }$ in the plane, and the perpendicular component ${\alpha }_{z}^{^{\prime\prime} }$, at the density of 1.5 · 10^−2^ *nm* ^−2^. The corresponding time fluctuations of these magnetization components are illustrated on the inset. We have identified on the same figure a range of concentration (grey) where the Knudsen number *K*_*n*_ = Λ/*l*, obtained from the water mean-free-path (Λ = 2.5 · 10^−^10 m in water) and the edge-to-edge inter-particle distance *l*, is not small enough to guaranty the absence of hydrodynamic effects. As shown on the same plot, this particular regime occurs in the restricted case where the particles are in contact (*ρ* ~ 10^−2^ *nm* ^−2^). As we will see later in this work, the damping by the liquid is so weak compared to the dipole-dipole interactions that the effect of the liquid can be completely neglected.

## Discussion

**Figure 3:**
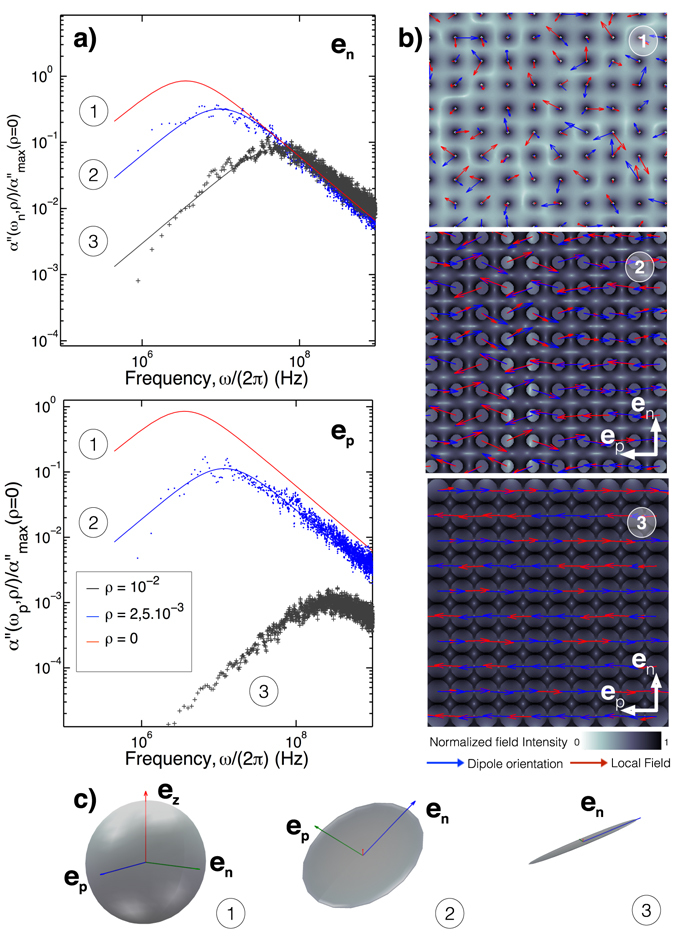
Susceptibility correlated with the local fields and dipole orientations. (**a**) Imaginary part of the in-plane magnetic susceptibilities ${\alpha }_{p}^{^{\prime\prime} }$ and ${\alpha }_{n}^{^{\prime\prime} }$ for the cases of no (1) (*ρ* = 0 *nm* ^−2^), medium (2) (*ρ* = 2.5 · 10^−3^ *nm* ^−2^) and strong (3) (*ρ* = 10^−2^ *nm* ^−2^) interactions. (**b**) 2D mapping of the dipoles orientation (blue arrows), local fields induced by the interactions (red arrows) and local field intensity (grayscale). (**c**) Ellipsoidal representation of the static magnetic susceptibility tensor for the three densities.

The dipole-dipole interactions (illustrated in Fig. 2) impact the amplitude of *α*′′(*ω*), which is dramatically decreased and the maximum of dissipation is shifted towards higher frequencies. In the time domain, orientational fluctuations of interacting dipoles are both accelerated and limited in amplitude compared to noninteracting dipoles. This is consistent with what has already been reported independently^15, 20, 28^. The planar distribution of the dipole lattice introduces an asymmetry between the in-plane and the cross-plane magnetization, which in turn influences their associated fluctuations: the latter being even faster and smaller than the former, less affected by the particle interactions. Surprisingly, regardless of the strength of the dipolar coupling, each component of the susceptibility still fits with a Debye function: an optimal frequency for dissipation thus corresponds to a single relaxation time. To estimate the mass concentration of nanoparticle in *g*/*l* from which the effect of the interactions becomes observable, we consider the mass density of iron oxide (5 · 10^3^ kg/m^3^). Dipolar effects occur as soon as the inter-particle distances become shorter than roughly three times the particle diameter (this correspondance between the dipolar energy cutoff and the particle diameter remains a good approximation even at other sizes, when accounting for a volume-dependent dipole moment). Considering the case of an isotropic particle distribution, the mass concentration is of the order of 250 g/L. Figure 2b presents the amplitude at the maximum of dissipation ${\alpha }_{max}^{^{\prime\prime} }(\rho )$ as a function of the density, for each component. These amplitudes are normalized by the corresponding values in the noninteracting case. Similarly, Fig. 2c displays the frequency of maximum dissipation 1/*τ* as a function of the MNP density. Importantly, above a density threshold of *ρ* = 4 · 10^−4^ *nm* ^−2^, the optimal frequency increases with density as *ρ* ^3/2^, similarly to the coupling energy *U* which scales as *d* ^−3^ = *ρ* ^3/2^. According to the Debye structure of the dissipation spectra, we deduce that the relaxation rate of rigid interacting dipoles includes an additional term which accounts for the interactions:

9 $${\tau }^{-1}={\tau }_{B}^{-1}+{\tau }_{U}^{-1}(\rho ),$$

with *τ*_*U*_(*ρ*) = ζ/*U*(*ρ*), this expression remarkably fits the statistical calculations presented in Fig. 2b,c for both the amplitude and frequency of maximum dissipation. However, we have noticed that when the interactions are accounted for, the susceptibility becomes anisotropic: each of the three eigenvalues of the imaginary part of *α* undergoes a different decrease of its amplitude in the directions of highest MNP density, as seen on Fig. 2b,c, for the two in-plane components (*x*, *y*). The spectra reported on Fig. 3a indicate that dipole-dipole interaction leads one of the two planar components (n or p) to undergo a singular effect from the interactions. To elucidate this particular feature, we have calculated both the average dipole orientation and the local field in the vicinity of each particle. By looking at the corresponding figure (Fig. 3b), we deduce that magnetic interactions tend to align the dipoles in particular directions, where the dipole density is the highest. In addition, it is observed that an antiferromagnetic phase order occurs as the thermal energy is dominated by that of the dipole pair (Fig. 3b-3)^25^. As depicted on Fig. 3a, this has for effect to saturate the magnetization along that direction (*e*_*p*_) while only the normal direction (*e*_*n*_) can be associated to a coupling with an external field.

**Figure 4:**
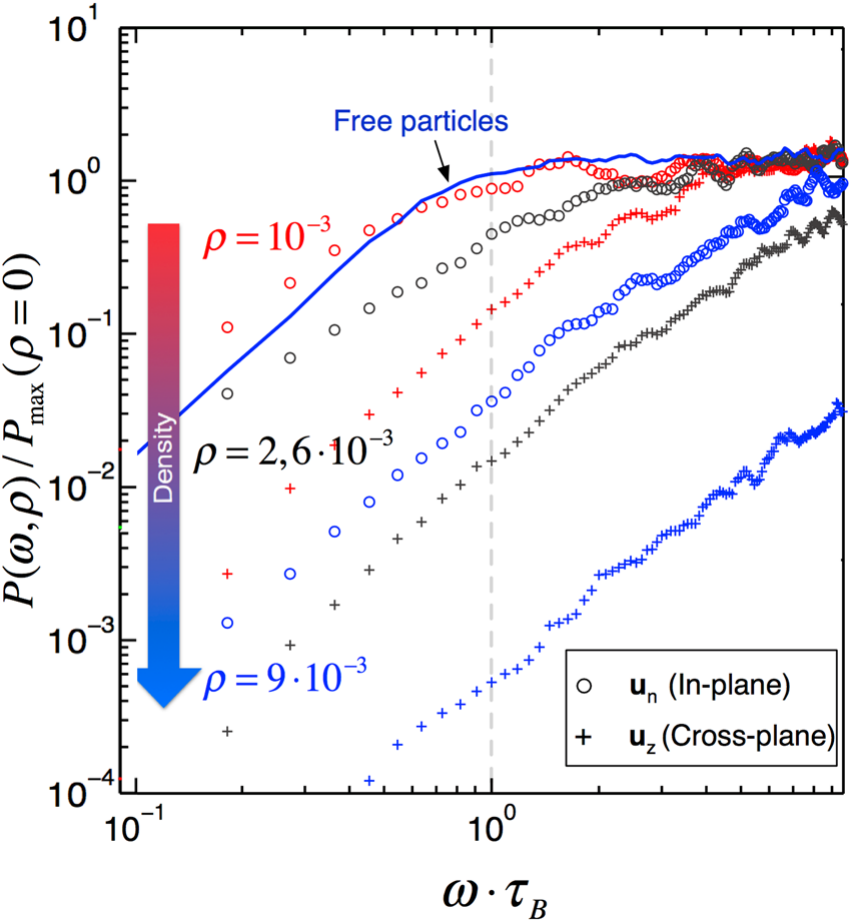
Influence of the dipole density on the power dissipated. Spectra of power dissipated at low (*ρ* = 10^−3^ *nm* ^−2^), medium (*ρ* = 2.6 · 10^−3^ *nm* ^−2^) and high density (*ρ* = 9.10^−3^ *nm* ^−2^), normalized by the maximum of power dissipated for noninteracting MNPs as a function of the frequency of the external field. The solid blue line corresponds to noninteracting MNPs. The maximum dissipated power is dramatically reduced by the interactions. For the sake of clarity, only the dominant in-plane and the cross-plane components are represented.

We conclude that interactions affect the susceptibility in two distinct ways. First, by orienting/reorganizing the dipoles in directions where the particle density is maximal. Here, we have two directions defining a square lattice in which the magnetization is confined. In addition to the confinement of the dipoles orientation, the ensemble of particles becomes increasingly less sensitive to the external field (the susceptibility saturates) as the result of an antiferromagnetic phase order that arises as the particle density grows. Again, this long-range ordering occurs along the direction where the particle density is high enough to counterbalance the thermal fluctuations. This can be further illustrated (Fig. 3-c): by associating each axis of an ellipsoid to the value of a component of the static magnetization (in-plane and cross-plane), one obtains a 3D representation of the system response. For instance, without interactions, the susceptibility tensor is spherical: the magnetization is isotropic. At weak interactions (Fig. 3c-2), the z-response vanishes, while the in-plane response dominates (with a quasi-degeneracy) and the static susceptibility can be represented by a disc. At high densities (Fig. 3c-3), the antiferromagnetic phase further restricts the magnetization of the system along a single in-plane direction (*e*_*n*_). We have finally estimated the power dissipated (*P* = *ωα*′′(*ω*)) for each frequency (Fig. 4). From this figure, it can be seen that for all densities, the energy dissipated in the liquid always increases with frequency until it saturates. The optimum is reached as soon as the period associated to the frequency of the external field matches the relaxation rate *τ*. Even with a weak dipole-dipole coupling, the possibility to heat-up a fluid with an external field is almost lost. On the remaining dominant absorption component, we see that for an inter-particle distance of the order of the particle diameter, the power dissipated drops from one to two orders of magnitude compared to the free particle case. These concluding remarks are of fundamental interest for hyperthermia applications where aggregates are very likely to occur. Experimental works have shown that when the particles are observed *in situ* in their biological environments, they are spatially confined. They are likely to behave in the way we have detailed here, that is strongly coupled in a highly anisotropic distribution.

In summary, the problem of estimating the collective effects (coupling) between magnetic nanoparticles has been solved by means of a statistical approach based on numerical simulations. Considering the equilibrium orientational fluctuations of magnetic dipoles, we have demonstrated that one can quantify the Brownian dissipation without approximations. Our work suggests that the interactions between magnetic dipoles have a dramatic impact on the fundamental mechanisms of dissipation: increasing the dipole pair energy enhances the Brownian relaxation rate and diminishes the magnitude of absorption. A microscopic analysis of this phenomena has revealed that in fact, interactions confine the orientations of the dipoles in directions of highest particle density. The interactions eventually lead to an antiferromagnetic ordering that completely saturates the response of the systems to any external excitation. Practically, these mechanisms are essential in the context of hyperthermia research, as it suggests that when the particle separation becomes smaller than a few diameters, the heating is achieved by tuning the external field at much higher frequencies. In addition, the amplitude of absorption diminishes importantly, while the control of the polarization of the field appears as an essential parameter to confort experimentally. A perspective of this work would be to carry this analysis *in situ*, in order to study the heat diffusion in more complex environments, where the intracellular confinement has affected the particle concentration.
